# Supplementary material for: Carbonate formation in salt dome cap rocks by microbial anaerobic oxidation of methane
Source: Nat Commun. 2019 Feb 18;10:808. doi: 10.1038/s41467-019-08687-z (PMC6379371; doi:10.1038/s41467-019-08687-z)
Supplement: Supplementary file 1 — Supplementary Information [file 41467_2019_8687_MOESM1_ESM.pdf]

Supplemental Information for:

Carbonate formation in salt dome cap rocks by microbial anaerobic oxidation of methane

Caesar et al.

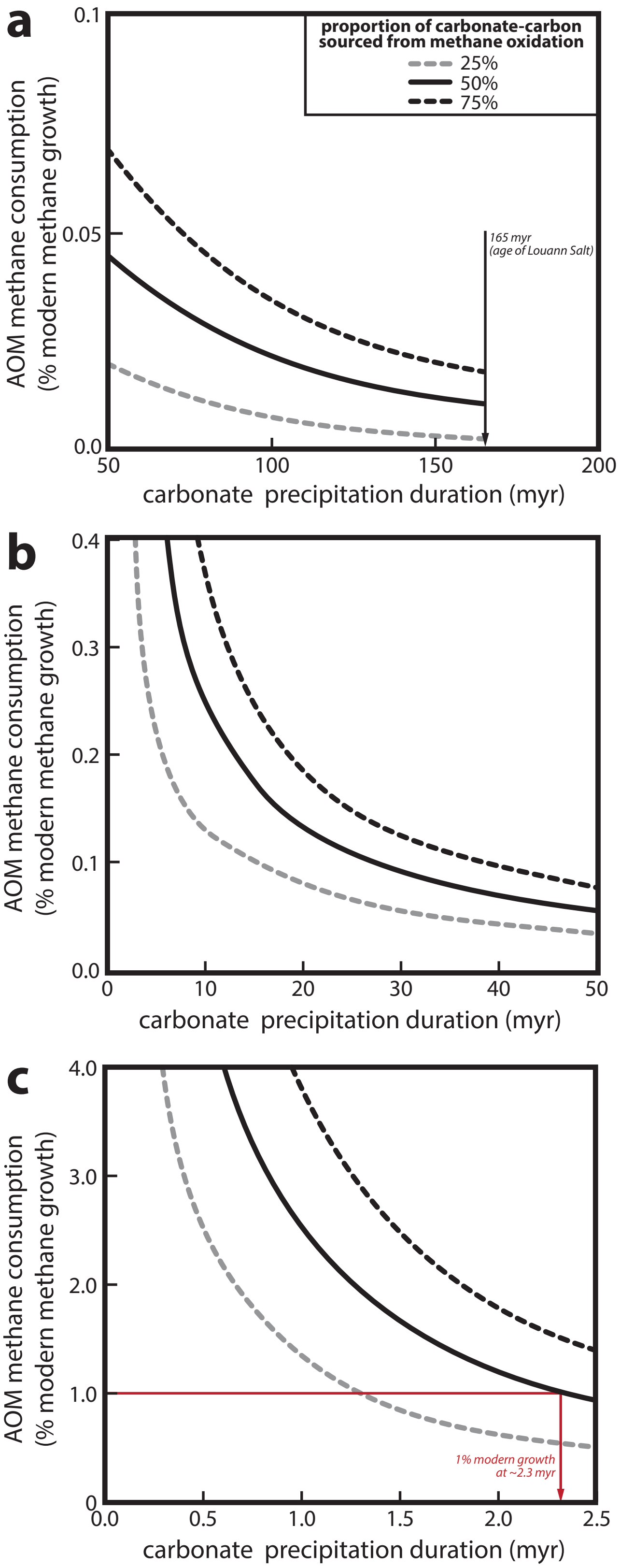

Supplementary Figure 1: Graphs depicting projected cap rock carbonate precipitation duration compared to recent atmospheric methane growth rates. Panels a, b and c reflect long, intermediate and short term AOM methane consumption possibilities, respectively. Methane growth rates have been approximated by Kirschke et al. to be ~6 Tg CH_4/yr[1] (or ~3.75 x 10^{11} mol C/yr). Approximately 1.8 x 10^{16} moles of carbon are preserved as carbonate cap rock in the Gulf of Mexico Basin (GMB) (see main text and references therein). Cap rock carbonate may have formed any time after deposition of the Louann Salt (deposited ~ 165 Ma[2]) and could still be occurring today (providing a maximum duration of 165 myr). Cap rock carbon is at least partially derived from methane (as discussed in main text) such that carbonate precipitation duration is proportional to methane oxidation duration (25, 50 and 75% methane oxidation-produced carbon possibilities are explored here). In order for methane oxidation (and subsequent carbonate precipitation with 50% methane carbon) to equal 1% of the atmospheric methane growth rate requires precipitation of all GMB cap rock carbonate in ~2.3 myr (refer to panel c).

Supplementary References

1 Kirschke, S. *et al.* Three decades of global methane sources and sinks. *Nature geoscience* **6**, 813 (2013).
2 Land, L., Kupecz, J. & Mack, L. Louann salt geochemistry (Gulf of Mexico sedimentary basin, USA): a preliminary synthesis. *Chemical Geology* **74**, 25-35 (1988).
